# Wine-processed radix scutellariae alleviates ARDS by regulating tryptophan metabolism through gut microbiota

**DOI:** 10.3389/fphar.2022.1104280

**Published:** 2023-01-05

**Authors:** Tingting Hu, Ying Zhu, Jing Zhu, Ming Yang, Yaqi Wang, Qin Zheng

**Affiliations:** ^1^ Jiangxi University of Chinese Medicine, Nanchang, China; ^2^ Blood Transfusion Department, First Affiliated Hospital of Gannan Medical University, Ganzhou, China

**Keywords:** acute respiratory distress syndrome, wine-processed radix scutellariae, gut microbiota, staphylococcal enterotoxin B, fecal metabolomics, 16S rrna

## Abstract

Acute respiratory distress syndrome (ARDS) is an acute and diffuse pulmonary inflammation, characterized by severe hypoxic respiratory failure caused by inflammatory tissue damage, which is a common cause of respiratory failure. Currently, there is no treatment available that can prevent or reverse the devastating effects caused by these conditions. The purpose of this study was to determine the effects of WRS on gut microbiota and the potential effect of gut microbiota on the treatment of lung disease by using a staphylococcal enterotoxin B (SEB)-induced ARDS model. The results showed that WRS could significantly reduce the pathological damage to lung and colon tissues and improve the lung and intestinal functions of ARDS mice. WRS was able to improve the level of cytokines in serum and lung tissue. Additionally, WRS could reverse the gut microbiota dysbiosis caused by SEB in ARDS mice. WRS increases the production of short-chain fatty acids (SCFAs) in the gut. This increase in SCFAs may lead to increased migration of SCFAs to the lungs and activation of free fatty acid receptors (FFAR) three and FFAR2 in lung epithelial cells, alleviating the symptoms of ARDS. Interestingly, WRS improves the faecal metabolite profiles in SEB-induced ARDS mice *via* tryptophan metabolism. On the basis of the component-target-metabolism strategy, baicalin, oroxylin A-7-O-glucuronide and skullcapflavon II were identified as the potential bioactive markers in WRS for the treatment of ARDS. Our study showed that WRS could ameliorate SEB-induced ARDS by regulating the structure of gut microbiota, increasing the production of SCFAs and modifying the faecal metabolite profiles through the lung-gut axis, and providing alternative treatment strategies for lung disease.

## Graphical Abstract

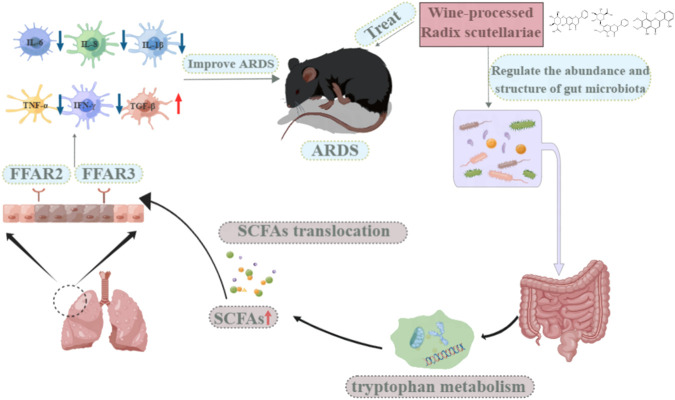

## Introduction

Acute respiratory distress syndrome (ARDS) is an acute respiratory failure that can be caused by several factors, and is characterized by respiratory distress, alveolar and capillary membrane damage, and hypoxia as the most prominent manifestations, with high mortality and morbidity rates (Batah and Fabro, 2021). The primary pathological feature of this disease is uncontrolled acute inflammation. Although, a number of anti-inflammatory therapies have been tested in clinical trials, including omega-3 fatty acids, neutrophil elastase inhibitors, corticosteroids, statins, beta-agonists, and granulocyte-macrophage colony-stimulating factors, none of these therapies resulted in significant reductions in mortality (Standiford and Ward, 2016). The most common cause of these cytokine storms is staphylococcal enterotoxin B (SEB), and no drugs are available to protect the host from the effects of SEB-mediated toxicity (Rubenfeld and Herridge, 2007; Nanchal and Truwit, 2018). Therefore, it is necessary to fully explore the pathophysiology of ARDS and develop new therapeutic approaches to prevent the development of this disease.

Although the physiological environment and functions of the digestive and respiratory systems are different, but they share the same embryonic origin and thus have similar physiological structures (Budden et al., 2017). Recent studies have shown that short-chain fatty acids (SCFAs), which are major metabolites of fiber fermentation and other indigestible carbohydrates, may have an impact on the health of these two organs/sites (den Besten et al., 2013). The SCFA molecules (e.g., acetate, butyrate and propionate) have been demonstrated to act as ligands for G-protein coupled receptors in the gut and have been shown to play an important role in the regulation of gut microbiota and host metabolism (Koh et al., 2016). Acetate-feeding has been shown to alleviate allergic airway disease (Thorburn et al., 2015). SCFAs could be transported to the lung along the gut-lung axis and regulate pulmonary immune tone (Liu et al., 2021). SCFAs may play an important role as mediators of the gut-lung axis. However, few studies have explored the role of gut bacteria and SCFAs in ARDS.

Radix scutellariae (RS), a well-known Traditional Chinese Medicine (TCM) for treating inflammation, is the dried roots of *Scutellaria baicalensis* Georgi (Chen et al., 2000; Chi et al., 2003; Woo et al., 2006). In addition to its medicinal effects, *S. baicalensis* leaves are rich in amino acids and selenium, which provide high-quality raw materials for the development of functional food (Sheng et al., 2009). The aerial parts of *S. lateriflora* have also been used as herbal tea and dietary supplements in European countries and the United States of America (Makino et al., 2008). According to the TCM theory, after being rice wine processed, WRS has an excellent therapeutic effect on clearing the heat in the lungs after being processed with rice wine (Fan and Li, 2000). Our previous research also showed that WRS has a better effect on the treatment of pneumonia than unpretreated RS (Hu et al., 2020). We also studied the variations in chemical constituents during rice wine processing. Furthermore, 10 components were identified as chemical markers to distinguish RS and WRS (Hu et al., 2020). However, among these 10 chemical markers, it is not clear which one is the bioactive ingredients are responsible for these functions and how they work.

To elucidate the effect of WRS on gut microbiota and the main active components of WRS in the treatment of ARDS, 16s rRNA sequencing and untargeted metabolomics were performed. This study is the first to demonstrate that WRS could alleviate ARDS through gut-lung axis and provides an alternative therapeutic strategy for the treatment of lung disease.

## Materials and methods

### Plant material

RS was purchased from Purechemland Inc. (Chengdu, Sichuan, China). Prof. Fei Ge (Jiangxi University of Chinese Medicine, China) checked and authenticated the samples. Voucher specimens (no. 21101601) were kept at the herbarium of Jiangxi University of Chinese Medicine, Nanchang, China.

### Sample preparation

50 g of RS decoction pieces were first macerated in 20% (10 ml) glutinous rice wine (Guyue Longshan, Shaoxing, Zhejiang, China) for 1 h, then, stir-fried for 18 min at a temperature of 120°C ± 10°C to obtain WRS.

After that 20 g of WRS was immersed in 200 ml water, followed by reflux extraction for 2 h. The extract was then filtered under vacuum to a concentration of 1 g/ml.

### Animals and drug administration

Female C57BL/6 mice (20 ± 2 g), 8 weeks of age, were obtained from Changzhou Cavens Experimental Animals Co., Ltd. (Changzhou, Jiangsu, China). Animal experiments were performed according to the animal experimentation guidelines, and the study protocols were approved by the animal ethics committee of Jiangxi University of Chinese Medicine, Nanchang, China (SCXK_2016-0010). Animals were housed in an animal laboratory room with temperature (22°C ± 2°C), humidity (50 ± 10%), and 12-h light/dark cycle. The animals were provided with pathogen-free food and water. Animals were acclimatized to their new environment 1 week prior to the experiment.

SEB was used in double doses to induce ARDS. Briefly, 25 μL of SEB (Toxin, Sarasota, FL, United States) was administered intranasally by micropipette at a dose of 5 μg per mouse. The second dose of SEB was administered intraperitoneally to the mice 2 hours after the first dose at a dose of 2 μg per animal.

After 1 week of adaptive feeding, mice were randomly grouped (10 mice per group) as normal control group (NC group), SEB-induced ARDS model group (SEB group), WRS treated groups (WRS group, 10 and 15 mg/kg) and the positive drug group dexamethasone (DXMS group, 5 mg/kg). As a preventive intervention, before SEB exposure, mice in WRS groups were orally administered with WRS extract for 14 days, while NC and SEB groups were treated with purified water simultaneously. Mice in the DXMS group were treated with purified water for the first 13 days, and then DXMS was administered intraperitoneally on the 14th day. Afterward, SEB was administered to mice in the WRS, SEB and DXMS groups. Tissue samples were harvested from the lungs, colon, thymus, and spleen 72 h following exposure to the second SEB dose.

### Treatment with antibiotics and faecal microbiota transplantation (FMT)

Healthy donor mice (*n* = 10) faeces were collected (Mohammed et al., 2020), diluted 1:10 (w/v) with saline, and homogenized for 1 min using a vertex mixer. Afterwards, particulate matter was removed by centrifuging the liquid slurry for 5 min at 200 × g. Afterwards, the supernatant was then aspirated in anaerobic conditions and immediately frozen, and was then administered to the mice, according to the experimental design, every day at a dose of 200 μL per animal.

Firstly, broad-spectrum antibiotics (ABX, containing 1 g/L of bacitracin, 0.5 g/L of gentamycin, 0.2 g/L of ciprofloxacin, 1 g/L of neomycin, 1 g/L of metronidazole, 0.5 g/L of ceftazidime, 1 g/L of penicillin, 2 g/L of streptomycin and 0.5 g/L of vancomycin) in drinking water were given to the recipient mice for 4 weeks to ensure that the endogenous microbiota was completely depleted. Next, they were transplanted daily with fresh faeces from healthy donor mice for 28 days. Then, the recipient mice received SEB exposure, as described above.

### Histopathology of lung tissues

The right upper lobe of mouse lung tissue (*n* = 3) was fixed in 4% paraformaldehyde solution (Meilunbio, Dalian, Liaoning, China) for 48 h at room temperature, and then embedded in paraffin and sectioned. The lung sections (5 μm of thickness) were stained with hematoxylin and eosin (H&E) (Solarbio, Beijing, China), and digital images of lung morphology were obtained using a Leica fluorescence microscope system (WETZLAR, Germany).

Lung tissue injuries were scored according to a four-stage grading system of pathology: no injury, within the normal range, 0; very slight, the change is just outside the range of change, one; mild, lesions can be observed, but not serious, two; moderate, the lesion is obvious and likely to be more severe, three; serious, the lesion is very serious, 4.

### Lung wet/dry ratio

The right lower lung lobe (*n* = 6) was removed, rinsed with normal saline, and then the excess water and blood on the surface of lung tissue were absorbed with filter paper, and weighted W). Dry mass D) was measured after drying at a 60°C incubator for 72 h. Wet/dry ratio (W/D) of lung tissue is calculated as wet weight g)/dry weight g) × 100%.

### Thymus and spleen index

After being cleaned with saline and excess water removed, the spleen and thymus (*n* = 10) were weighed accurately to evaluate the organ index of mice. The formula used for the calculation of organ index is organ weight (mg)/mouse weight g)×100%.

### Real-time qPCR

Extraction of total RNA from lung tissue (*n* = 6–8) was performed with TRIzol reagent (Aidlab, Beijing, China). RNA concentration was equilibrated and transformed into cDNA with the kit (HiScript reverse transcription, Vazyme, Nanjing, China). BIO-RAD Real-Time System detection system was used for qPCR analysis. The expression of target genes and β-actin was calculated by 2^−ΔΔCT^. Primer sequences of the target gene used in this experiment are listed in Table 1.

**TABLE 1:** Primer sequences of the target gene.

target gene	Forward	Reverse
β-actin	5′-TCA​TCA​CTA​TTG​GCA​ACG​AGC-3′	5′-AAC​AGT​CCG​CCT​AGA​AGC​AC-3′
IL-6	5′-TGT​AAC​TGG​CCT​GCA​GTA​GC-3′	5′-CTT​TCC​CTC​ACC​CTA​GCA​GC-3′
IL-8	5′-GTA​GTT​GTG​CTC​GCT​CTC​ATT-3′	5′-GTT​CGC​TTT​TCT​CAG​CAG​AGT​TTA-3′
IL-1β	5′-ATG​AAA​GAC​CTC​AGT​GCG​GG-3′	5′-AAG​GGG​ACA​TTA​GGC​AGC​AC-3′
TNF-α	5′-ATA​GCA​AAT​CGG​CTG​ACG​GT-3′	5′-AGC​CGA​TGG​GTT​GTA​CCT​TG-3′
IFN-γ	5′-CGG​CAC​AGT​CAT​TGA​AAG​CC-3′	5′-TAG​CAA​CGT​AGC​ACC​CCA​TC-3′
TGF-β	5′-AAA​ACG​AAC​CAG​CGA​ACG​T-3′	5′-GAG​GCA​GCG​TTT​TTC​GTG​TT-3′
FFAR3	5′-GCA​GGT​CCG​AAA​TGG​TCA​G-3′	5′-ACC​TGT​TGG​TGT​TCC​TCG​TG-3′
FFAR2	5′-CAC​CCC​TGT​CCA​TCT​TGG​TC-3′	5′-TAC​TGA​TCC​GCA​ATC​CTG​CC-3′

### ELISA of cytokines

Serum samples (*n* = 6–10) were used to detect cytokines, including of transforming growth factor-beta (TGF-β), interferon-gama (IFN-γ) and tumor necrosis factor-alpha (TNF-α). These cytokine levels were determined according to the instructions provided in the ELISA kits (Meimian, Yancheng, Jiangsu, China).

### Faecal metabolomics

20 mg of faeces were homogenized with 120 μL of methanol, and centrifuged at 4°C and 14,000 r/min for 10 min. Then the supernatant was immediately transferred and filtered through a 0.22 µm membrane before analyzing by a liquid chromatograph-mass spectrometer (LC-MS). To ensure the data quality of metabolic profiling, we prepared quality control samples (*n* = 8).

We used a Phenomenex Kinetex C18 column (100 mm × 2.1 mm, 2.6 μm) and Triple TOFTM 5600 (AB Sciex, Foster City, CA, United States of America) LC-MS with DuoSprayTM ion source to perform the liquid chromatographic separation. The mobile phase was 0.1% formic acid in water (v/v, A)-acetonitrile B) at a 0.3 ml/min flow rate and 40°C column temperature. Gradient elution was applied as follows: 0–4 min, 5–25% B; 4–10 min, 25–45% B; 10–22 min, 45–95% B. Mass spectrometer parameters: drying gas, N_2_; gas temperature, 500°C; ion spray voltage, 5500 V; collision energy, 20 eV; declustering potential voltage, 100 or -100 V; sheath and auxiliary gas flow rate, 55 psi; scanning range, 50–1250 m/z were applied.

Peakview (ver 1.2), Markerview (ver 1.3.1) and SIMCA-P 14.0 software were used to process UPLC-MS data. After data preprocessing (baseline correction, peak alignment and scaling), metabolites were identified by KEGG (http://www.kegg.ca/) and Human Metabolome Database (HMDB, http://www.hmdb.ca/). Metabolic pathway analysis and potential biomarker screening were performed through Metaboanalyst 5.0 (http://www.MetaboAnalyst.ca).

### 16s rRNA sequencing

Faecal genomic DNA (*n* = 4–6) was extracted with a DNA kit and quantified by agarose gel electrophoresis. We used specific primer 341 F (5′-CCTACGGGRBGCASCAG-3′) and 806R (5′-GGACTACHVGGGTWTCTAAT-3′) to amplify the V3-V4 regions of the 16s rRNA genes. We used a Qiagen Gel extraction kit (Qiagen, Germany) to purify the PCR products. We generated the sequencing libraries following the recommendations of the manufacturer of TruSeq^®^ DNA PCR-Free sample preparation kit (Illumina, United States). We sequenced this library using a paired-end sequencing strategy on the Illumina NovaSeq6000 platform (Illumina, California, United States).

### SCFAs measurement

To preserve volatile SCFAs in faeces, sample extraction was performed at 4°C. 30 mg of faeces samples (*n* = 4–5) and 1 ml 0.005 M of NaOH aqueous solution (containing 5 μg/ml 2-ethylbutyric acid as an internal standard) were homogenized and centrifuged for 10 min at 14000 rpm. Supernatants were treated through the derivatization reaction method as previously described (Singh et al., 2019).

Gas chromatography-mass spectrometry (GC-MS) was performed using an Agilent 7890A gas chromatography system and an Agilent 5975C mass spectrometric detector (MSD, Agilent Technologies, Santa Clara, CA, United States). 1 μL of each sample derivative was injected into an HP-5ms capillary column (30.0 m × 250 µm i. d., 0.25 µm film thickness, Agilent J and W Scientific, Folsom, CA, United States). Helium was used as a carrier gas at a 1 ml/min flow rate, operating in a 10:1 split mode ratio and 2.5 min solvent delay time. The oven temperature program was set as follows: 50°C for 2 min, 70°C at a rate of 10°C/min, 90°C at a rate of 3°C/min, 110°C at a rate of 10°C/min, 290°C at a rate of 20°C/min; 260°C front inlets, 280°C transfer line, and 230°C electron impact ion source; 70 eV electron energy, and selected ion monitoring (SIM) mode. Agilent MSD Chemstation (Santa Clara, CA, United States) was used for the data analysis.

### Predictive analysis of active constituents in WRS

The targets of the different chemical components of WRS were retrieved and collected from two online target prediction platforms: Swiss Target Prediction (http://www.swisstargetprediction.ch/) and Stitch (http://stitch.embl.de/) database. Subsequently, intersection processing was performed with the targets derived from the metabolic pathway to screen the common targets of the component and metabolism. The number of targets involved in the component accounted for 100% of the total number of targets in the corresponding pathway were selected as the main active constituents.

### Statistical analysis

All data requiring statistical calculations were processed using GraphPad Prism (version 7.0, San Diego, United States) and presented as mean ± SD. Statistical analysis was performed using Student’s t-test and one-way ANOVA. Differential metabolites were filtered by variable importance in the projection (VIP) > 1.0 and *p* < 0.05.

## Results

### WRS protects against ARDS in response to the SEB challenge

**FIGURE 1:**
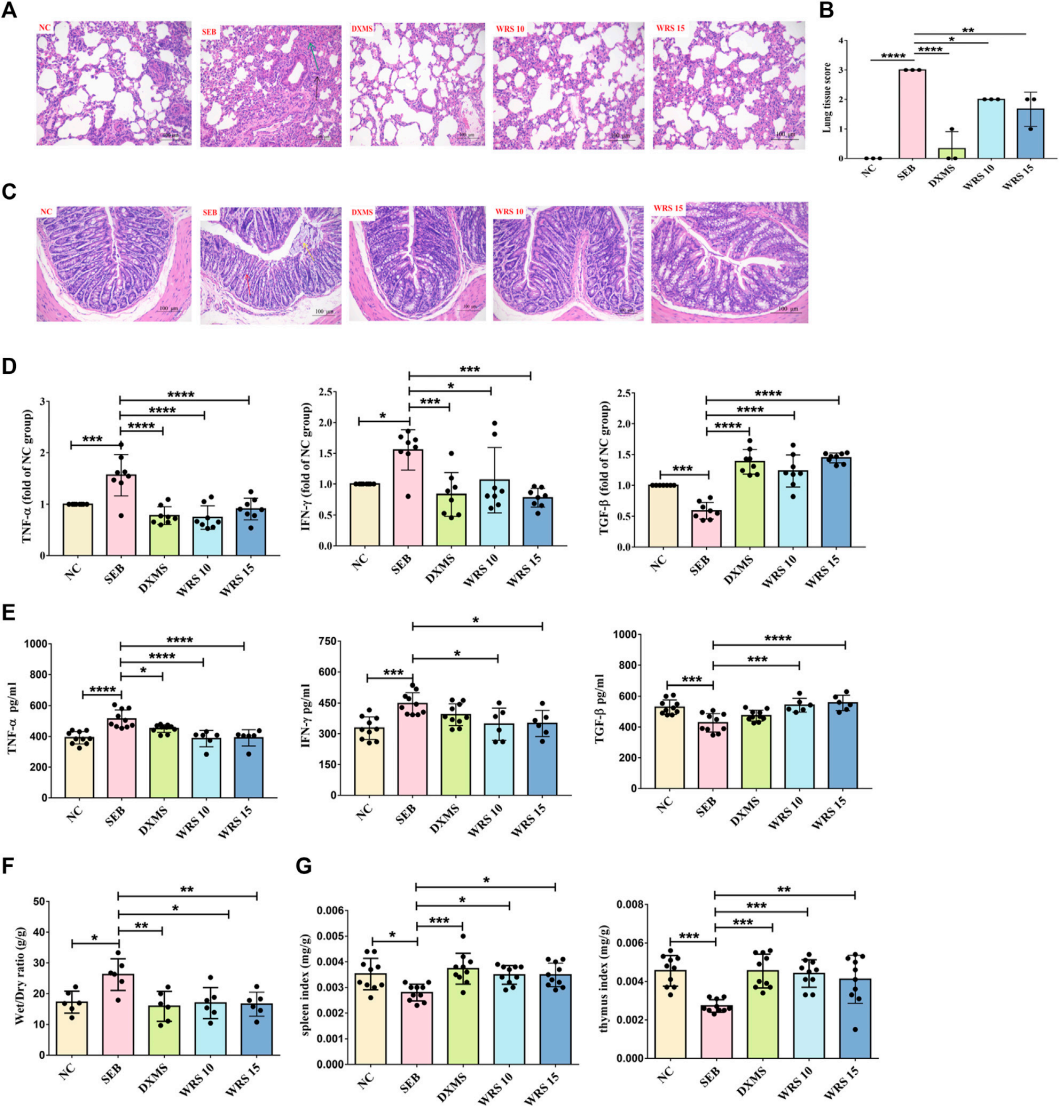
WRS reduces symptoms and inflammation associated with ARDS. **(A)** The pathological changes of paraffin sections of lung tissues were measured by H&E staining. Scale = 200 μm. **(B)** Lung tissue injury scores among groups (*n* = 3). **(C)** The pathological changes of paraffin sections of colon tissues were measured by H&E staining. Scale = 200 μm. **(D)** Levels of three cytokines from the lung tissue (*n* = 8). **(E)** Levels of three cytokines from the serum (*n* = 6–10). **(F)** The lung wet/dry ratio (*n* = 6). **(G)** The organ index of spleen and thymus (*n* = 10). Data have been presented as means ± SD. Statistical analysis was carried out using one-way ANOVA. *p < 0.05, **p < 0.01, ***p < 0.001, ****p < 0.0001.

SEB, a superantigen, could cause acute inflammation. The current study investigated whether WRS treatment would improve acute lung inflammation prior to SEB sensitization. Pathological changes in lung tissue are a major feature of mouse models, therefore ARDA mice model was developed and treated according to the treatment protocols mentioned earlier in the experiment section. In the NC group, the lung tissue sections of mice were clear, and no obvious pathological changes were observed (Figure 1A). After SEB exposure, lung tissue structure was damaged, the alveolar walls were thickened, and there was marked infiltration of inflammatory cells (Figure 1A). Interestingly, after WRS treatment, lung tissue structure was substantially improved and inflammatory cell infiltration was significantly reduced (Figure 1A). A four-stage grading system of the pathology of the lung histopathological changes indicated that either treated with 10 or 15 mg/kg WRS improved the progress of lung tissue injuries (Figure 1B).

Many studies have shown that the primitive foregut is the embryonic origin of respiratory and gastrointestinal epithelial cells (Ramalho-Santos et al., 2000; Shu et al., 2007). This similarity is thought to be partly responsible for lung-gut crosstalk during inflammation (Keely et al., 2012). We also observed the pathological sections of colon tissue, as shown in Figure 1C. SEB group showed surface epithelial erosion, crypt destruction, muscularis mucosa destruction, submucosa edema, and inflammatory cell infiltration. However, these symptoms were greatly relieved in WRS-treated mice.

Prevention or suppression of cytokine storm may be one of the strategies for treating severe pneumonia patients. To determine if WRS could have a better effect on calming cytokine storm, the levels of pro-inflammatory cytokines (TNF-α, IFN-γ) and anti-inflammatory mediator (TGF-β) in serum and lung tissue were measured (Figures 1D, E). These cytokines were significantly improved after WRS treatment compared with the SEB group. Encouragingly, WRS had a remarkable regulatory effect which was similar to or even better than the positive drug DXMS, indicating that WRS efficiently inhibited cytokine storms.

WRS significantly reduced the lung edema (lung wet/dry ratio) compared with the SEB group (Figure 1F). The spleen and thymus are the main immune organs that drive most immune responses (Checker et al., 2008). WRS significantly raised the thymus and spleen indices, indicating an enhancement of immune function by WRS (Figure 1G). These results together demonstrated that WRS ameliorated the inflammatory response and possibly induced lung immune hemostasis.

### WRS alleviates ARDS-induced gut dysbiosis

**FIGURE 2:**
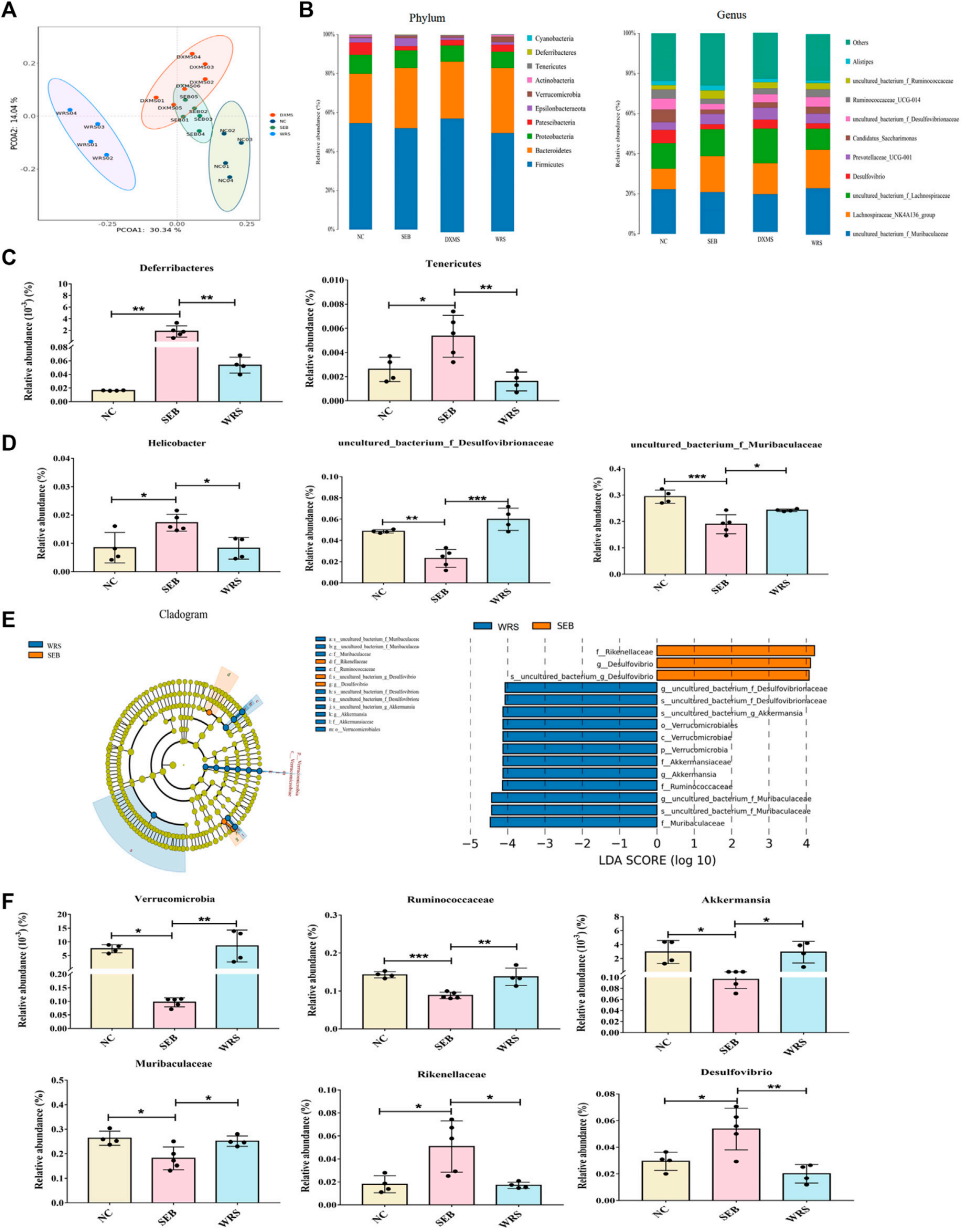
WRS altered the gut microbiota community composition of mice with SEB infection. **(A)** Principal co-ordinator analysis. **(B)** Top 10 most abundant bacterial at the phylum level and genus level. **(C,D)** Representative histogram of the gut microbiota at the phylum level and genus level. **(E)** LEfSe and cladogram analysis. **(F)** Relative abundance of key bacterial. Data have been presented as means ± SD (*n* = 4–6). Statistical analysis was carried out using one-way ANOVA. *p < 0.05, **p < 0.01, ***p < 0.001.

Gut microbiota plays an important role in the evolution of lung disease, and microbiota modulation is a potential therapeutic approach to prevent ARDS (Dickson et al., 2016; Sultan et al., 2021). We investigated the role of WRS (15 mg/kg) in regulating microbial dysregulation in ARDS. 16s rRNA-seq analysis revealed that more than 99.9% of the sequence exhibited good coverage values, indicating the sequencing results’ reliability. WRS significantly altered the composition and relative abundance of faecal microbiota, according to the principal co-ordinator analysis (PCoA) and *a*-diversity index (Figure 2A, Supplementary Table S1). 10 most abundant phylum and genus were presented in Figure 2B. At the phylum level, Deferribacteres and Tenericutes were enriched in SEB group. At the genus level, *Helicobacter* were enriched in the SEB group, while Desulfovibrionaceae and Muribaculaceae were more abundant in the WRS group (Figures 2B–D).

To further determine the dominant microbial species, linear discriminant analysis (LDA) effect size (LEfSe) analysis was used. The relative abundance of Verrucomicrobia at the phylum level, Ruminococcaceae and Muribaculaceae at the family level, *Akkermansia* at the genus level was remarkably increased after giving WRS, while decreasing the abundance of Rikenellaceae at the family level and *Desulfovibrio* at the genus level from ARDS mice (Figures 2E, F). These data demonstrated that alterations in microbial composition and function caused by SEB-induced ARDS (Supplementary Figures S1–4) could be effectively regulated with WRS treatment.

### WRS enhances SCFAs production and FFAR2, FFAR3 expression

**FIGURE 3:**
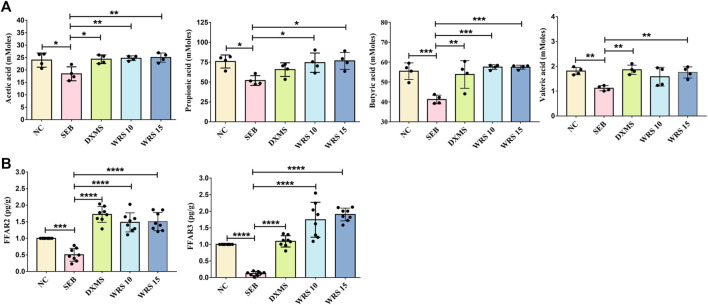
Effects of WRS on short-chain fatty acids in mice. **(A)** Change of content of acetic acid, propionic acid, butyric acid and valeric acid (n = 4). **(B)** Change of mRNA expression of FFAR3 and FFAR2 (*n* = 8). Data have been presented as means ± SD. Statistical analysis was carried out using one-way ANOVA. *p < 0.05, **p < 0.01, ***p < 0.001, ****p < 0.0001.

As major bacterial metabolites, SCFAs interact with receptors on host cells that can activate or inhibit signaling pathways and regulate multiple metabolic pathways in the gut and at distances. To clarify the correlation between SCFAs and ARDS, we measured SCFAs concentrations in the faeces by GC-MS. As shown in Figure 3A, acetic acid, propionic acid, butyric acid and valeric acid were significantly increased after WRS treatment compared with the SEB group (Figure 3A). Recent studies have shown that SCFAs modulate epithelial cells or neutrophil immune responses that depend on the sensing receptors, FFAR3 and FFAR2 (Kim et al., 2013; Zhao, 2013). However, the effect of WRS on FFAR3/FFAR2 has not been reported before. Consequently, we detected the mRNA expression of FFAR3 and FFAR2 in lung tissue by qRT-PCR. As shown in Figure 3B, the mRNA expression of FFAR3 and FFAR2 was strikingly lowered upon SEB compared with the NC group, and the reduction of FFAR3 and FFAR2 mRNA expression by SEB was significantly reversed by WRS treatment (Figure 3B). Our results suggested that WRS treatment increased production of SCFAs in the gut, and then the increased the SCFAs may migrate to the lung and activate FFAR3 and FFAR2 in lung epithelial cells to fight against ARDS infection.

### Effects of faecal microbiota transplantation on SEB-induced ARDS

**FIGURE 4:**
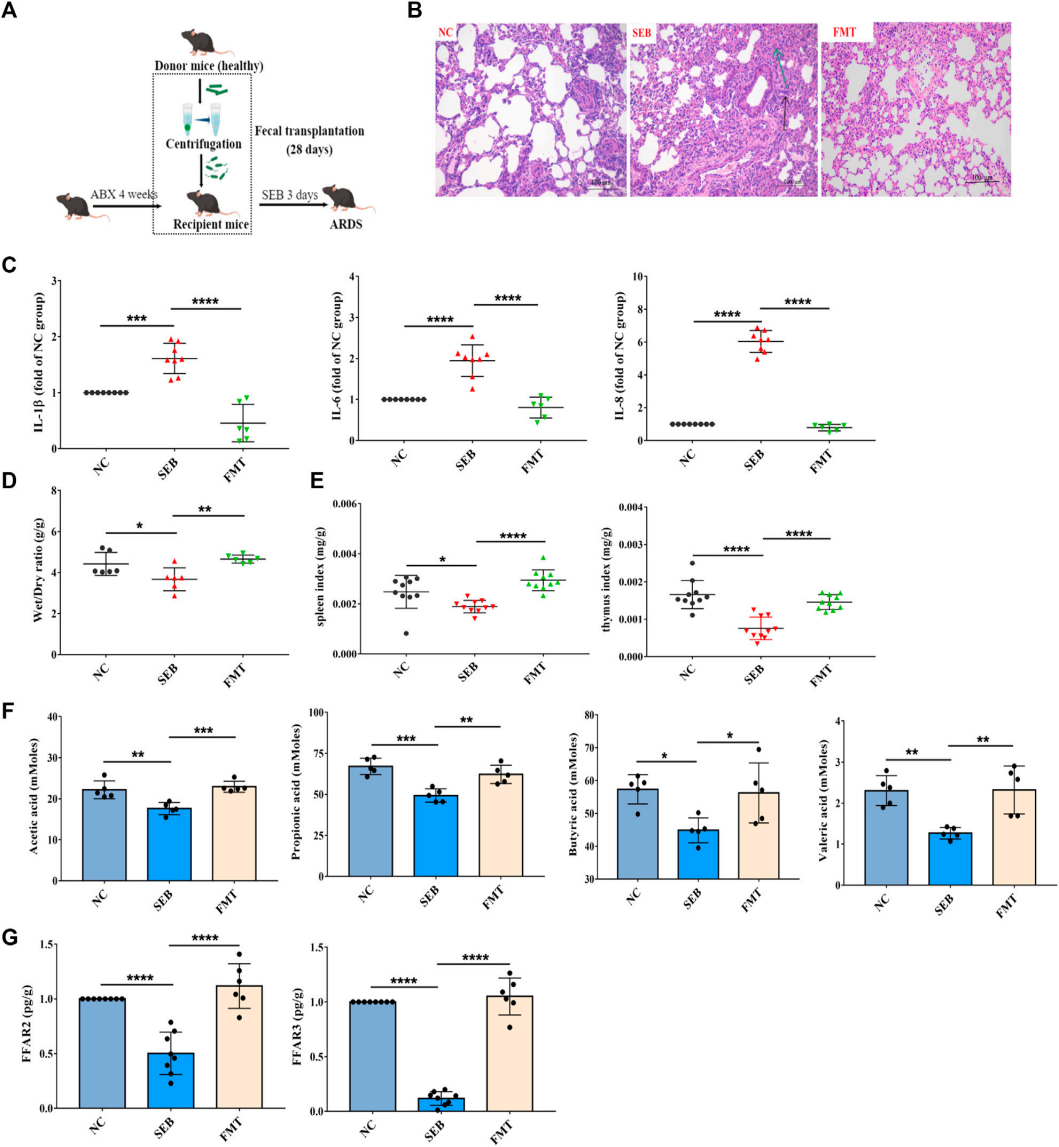
Effects of faecal microbiota transplantation (FMT) on microbiota reconstitution and ARDS pathogenesis. **(A)** Schematic diagram of FMT in ARDS mice. **(B)** The pathological changes of paraffin sections of lung tissues were measured by H&E staining. Scale = 200 μm. **(C)** Levels of three cytokines from the lung tissue (*n* = 6–8). **(D)** The lung wet/dry ratio (*n* = 6). **(E)** The organ index of spleen and thymus (*n* = 10). **(F)** Change of content of acetic acid, propionic acid, butyric acid and valeric acid in the feces (*n* = 5). **(G)** Change of mRNA expression of FFAR3 and FFAR2 (*n* = 6–8). Data have been presented as means ± SD. Statistical analysis was carried out using one-way ANOVA. *p < 0.05, **p < 0.01, ***p < 0.001, ****p < 0.0001.

To confirm whether the changed gut microbiota after WRS treatment was responsible for the alleviation of ARDS, FMT was used to test the role of gut microbiota in the treatment of ARDS. After being treated with a cocktail of antibiotics for 4 weeks, gut microbes were effectively depleted (Supplementary Figures S5, 6). Microbiota from healthy mice was transplanted into recipient ABX mice. After 4 weeks of colonization, then the recipient mice were exposed to SEB (Figure 4A). As shown in Figure 4, FMT treatment demonstrated similar lung inflammation protective effects as observed in WRS groups. Recipient mice showed improved lung tissue structure, inflammatory factors and organ index (Figures 4B–E). Besides, SCFA concentrations and FFAR3 and FFAR2 mRNA expression were also restored (Figures 4F,G). Together, these results demonstrated that the homeostasis of gut microbes played an important role in protecting lung health, and WRS alleviates ARDS by reconstructing the microbial microenvironment.

### WRS improves the faecal metabolite profiles of mice with SEB-induced ARDS

**FIGURE 5:**
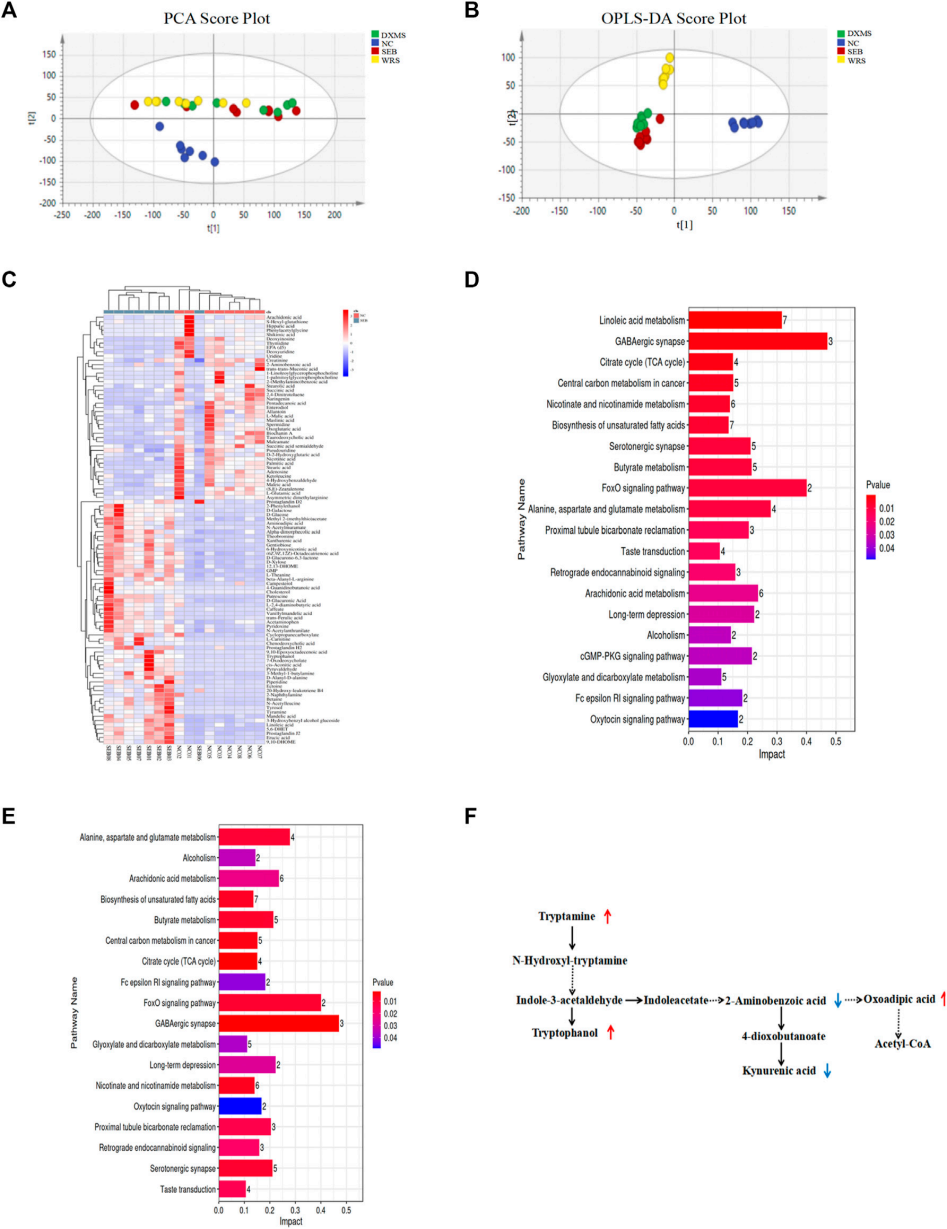
Effect of WRS on fecal metabolites in mice with SEB infection. **(A)** PCA plot. **(B)** OPLS-DA plot. **(C)** Differential metabolites between NC group and SEB group. **(D)** Changes of metabolic pathway enrichment between NC and SEB groups. **(E)** Changes of metabolic pathway enrichment between SEB and WRS groups. **(F)** Correlation among differential metabolites involved in tryptophan metabolism. Data have been presented as means ± SD (*n* = 8). Statistical analysis was carried out using one-way ANOVA. *p < 0.05, **p < 0.01, ***p < 0.001.

The effect of gut microorganisms on the host is closely related to the complex interactions of a series of host-microbial metabolic axes (Zeng et al., 2020). Untargeted metabolomic analysis of faeces samples by LC-MS was performed to assess the metabolic role of WRS-reconstituted (15 mg/kg) gut microbiota. The results of quality control samples are shown in Supplementary Table S2. PCA plot showed that metabolite clustering was evident among NC, WRS, DXMS and SEB groups by PCA plot (Figure 5A). Orthogonal partial least squares-discriminant analysis (OPLS-DA) also revealed an obvious separation among these groups, with R^2^X, R^2^Y and Q^2^ being 0.588, 0.905 and 0.624 between NC and SEB groups (Figure 5B). SEB treatment was sufficient to induce a wide range of changes in metabolites, with 74 and 44 metabolites significantly up-regulated and down-regulated, respectively (Figure 5C). Notably, after administration of WRS, some metabolites in the SEB group were regulated, and 12 metabolite changes induced by SEB were eliminated (5 up-regulated and seven down-regulated) (Table 2).

**TABLE 2:** 12 significantly different metabolites in the feces of mice between SEB group and WRS group.

Peak no.	name	mz	rt	Formula	KEGG	VIP	Trend of WRS/SEB
1	2-(Methylamino)benzoic acid	134.0589	378.00	C8H9NO2	C03005	11.18	↑*
2	2-Aminobenzoic acid	137.0447	86.12	C7H7NO2	C00108	1.76	↑*
3	7-Oxodeoxycholate	387.2569	833.48	C24H38O5	C04643	4.82	↓*
4	Citric acid	191.0186	105.22	C6H8O7	C00158	1.73	↑*
5	l-Carnitine	144.1005	115.58	C7H15NO3	C00318	2.40	↓*
6	l-Glutamic acid	148.0589	80.53	C5H9NO4	C00025	1.85	↑*
7	Maleic acid	115.0396	180.21	C4H4O4	C01384	1.01	↓*
8	Maslinic acid	471.3374	1157.95	C30H48O4	C16939	2.18	↓*
9	N-Methylhydantoin	115.0530	310.55	C4H6N2O2	C02565	1.17	↓***
10	Prostaglandin D2	351.2181	645.29	C20H32O5	C00696	2.53	↓*
11	Thymidine	241.0814	167.17	C10H14N2O5	C00214	8.55	↓*
12	trans-trans-Muconic acid	143.0384	310.02	C6H6O4	C02480	1.93	↑***

Furthermore, differential metabolites with a fold change larger than 1.2 or less than 0.8 were analyzed for screening potential metabolic pathways by MetaboAnalyst (*p* < 0.05, impact value >0.01). Between healthy and SEB mice, as shown in Figure 5D, 26 metabolic pathways were identified as significant metabolic pathways (Figure 5D). Among these 26 potential pathways, WRS mainly regulated amino acid metabolism, including tryptophan metabolism, lysine degradation and arginine biosynthesis (Figure 5E). The most enriched pathway for differential metabolites with the largest impact value was the tryptophan metabolism, in which 2-aminobenzoic acid, oxoadipic acid, tryptamine, tryptophanol and kynurenic acid were enriched (Figure 5F).

### Screening of potential bioactive markers in WRS on ARDS

**FIGURE 6:**
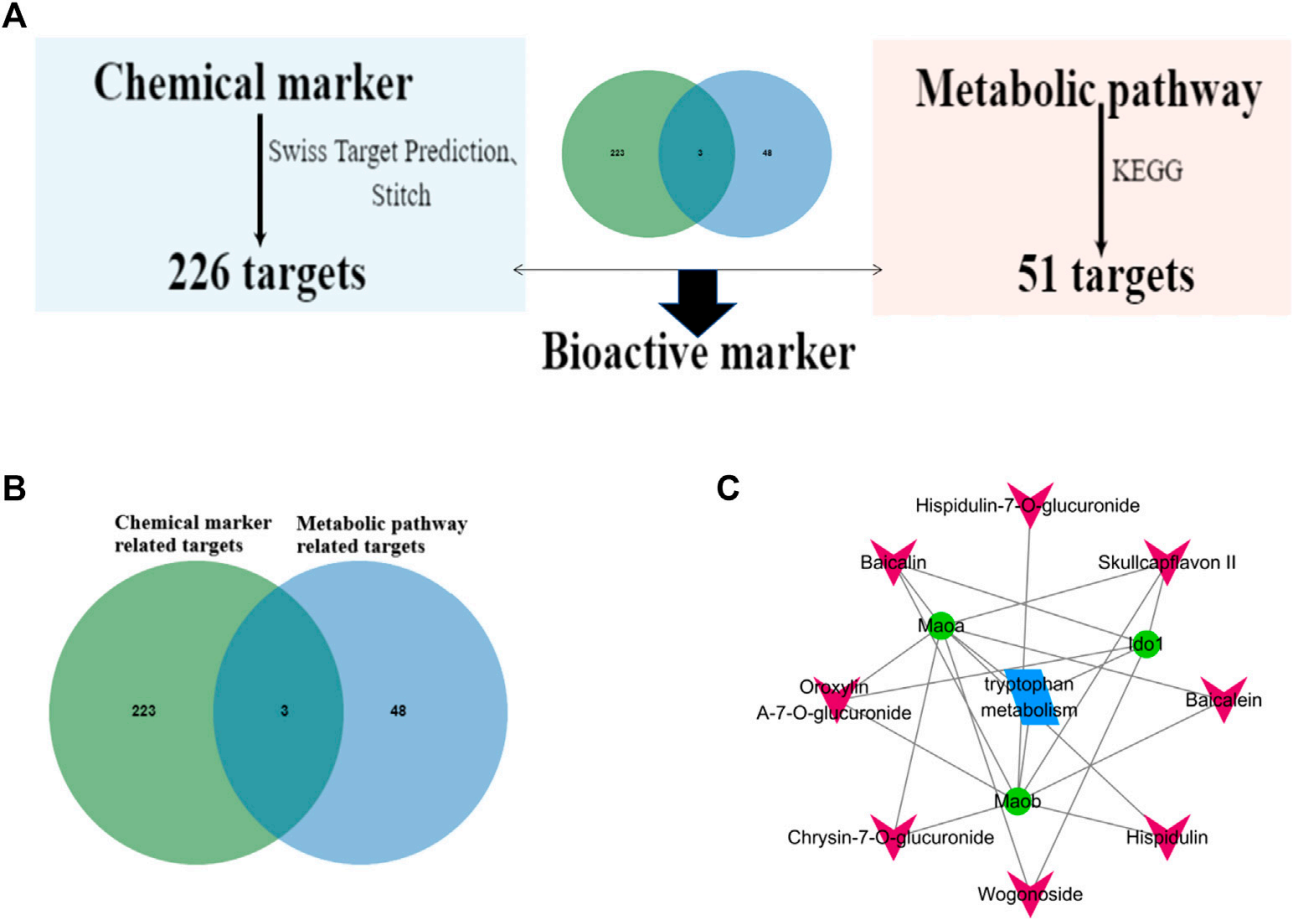
Screening of potential bioactive markers in WRS on ARDS. **(A)** Flowchart of screening bioactive markers of WRS. **(B)** Cross targets of chemical markers and metabolic pathway related targets. **(C)** WRS chemical markers-target-pathway network.

According to our previous research, 10 compounds were identified as chemical markers in WRS (Supplementary Table S3; Hu et al., 2020). Among the 10 chemical markers, 226 gene targets were retrieved from the Swiss Target and Stitch Prediction database. Then, gene intersections were generated by mapping the targets of the 10 chemical markers with the selected tryptophan metabolism using the KEGG database. Consequently, three targets of eight components in WRS associated with tryptophan metabolism were screened out (Figure 6B). WRS constituent-target-pathway network was constructed to reveal the intersections of the three target symbols using Cytoscape software (Figure 6C). If the target number involved in each component accounted for 100% of the target number of all eight chemical markers, it was selected as bioactive marker. Finally, three potential bioactive markers (baicalin, oroxylin A-7-O-glucuronide, skullcapflavon II) in WRS were screened (Table 3). In this study, we found for the first time that WRS could exert a profound therapeutic effect on ARDS by acting on MAoa, MAob, Ido one through baicalin, oroxylin A-7-O-glucuronide and skullcapflavon II.

**TABLE 3:** Network pharmacology integrated metabolomics.

pathway	Chemical markers	“Pathway-component” targets	Number of targets	Proportion of the total number of targets (%)
tryptophan metabolism	Baicalin	Maob,Ido1,Maoa	3	100
	Oroxylin A-7-O-glucuronide	Maob,Ido1,Maoa	3	100
	Chrysin-7-O-glucuronide	Maob, Maoa	2	66.67
	Wogonoside	Ido1,Maoa	2	66.67
	Hispidulin	Maob, Maoa	2	66.67
	Baicalein	Maob, Maoa	2	66.67
	Hispidulin-7-O-glucuronide	Maob	1	33.33
	Skullcapflavon II	Maob,Ido1,Maoa	3	100

## Discussion

Presently, ARDS has a mortality rate of 30%–40%, and existing therapeutic approaches are insufficient to prevent the condition from becoming more serious (Zambon and Vincent, 2008). Many studies have found that gut microbiota is critical in regulating inflammation. However, it is unknown how the resident microbiota influences the volution of ARDS.

Previous studies showed that WRS has an anti-inflammatory effect that can significantly decrease the levels of pro-inflammatory cytokines, including nitric oxide, IL-6, TNF-α, and IL-8 (Hu et al., 2020). In our study, we tried to investigate the effect of WRS on the gut microbiota and elucidate how the ecological imbalance affects ARDS. Our findings showed that WRS relieves SEB-induced lung inflammation is associated with its modulation of microbiota dysbiosis. Increased SCFAs may play an important role as transport mediators of the lung-gut axis.

The gut microbiota evolved and existed in a symbiotic relationship with the host, contributing to the regulation of the intestinal barrier, immune response, development and maintenance of energy metabolism and other physiological functions (Gilbert et al., 2018). There is evidence that the gut microbiota has a powerful effect on reducing lung inflammation, and considerable alterations in the gut microbiota can be observed in animal models (Sultan et al., 2021; Tang et al., 2021). Stimulation of pattern recognition receptors including Toll-like receptors and NOD-like receptors can be induced by microbial molecules and lead to the induction of IL-1β expression (Turner et al., 2014). Our study demonstrated that the community composition of the microbiota from the SEB group was dramatically different from that of the WRS group. Additionally, FMT from healthy mice relieved ARDS in recipient mice, indicating that the gut microbiota plays a key role in the therapeutic effects of WRS. *Akkermansia muciniphila*, has been shown to induce regulated immunity in mice and positively affect diseases mediated by low-grade chronic inflammation (Hansen et al., 2012; Everard et al., 2013; Shin et al., 2014). Ruminococcaceae is positively correlated with mRNA expression of tight junction proteins, pro-inflammatory cytokines and SCFA receptors (Dong et al., 2020). Ruminococcaceae is also positively correlated with Treg cell counts (Han et al., 2018). Muribaculaceae abundance is an important predictor of SCFAs content in the gut (Smith et al., 2019). Metagenomics results showed that Muribaculaceae possess fermentation pathways to produce acetate, propionate and succinate by degrading of dietary polysaccharides (Ormerod et al., 2016; Lagkouvardos et al., 2019). Muribaculaceae were negatively correlated with inflammation due to their ability to promote the production of SCFAs and improve immune cell function (Wu et al., 2019; Li et al., 2020; Shang et al., 2021). Rikenellaceae is related to intestinal inflammation (Kim et al., 2012; Zenewicz et al., 2013). *Desulfovibrio* is an inflammation-related pathogen that can reduce sulfate to the cytotoxic compound hydrogen sulfide (Lennon et al., 2014). It not only promotes the production of lipopolysaccharide, but also degrades and metabolizes SCFAs (Chen et al., 2019). The above results indicated that the effects of WRS on pulmonary inflammatory responses might be closely linked to the regulation of the gut microbiota.

Alterations in microbiota homeostasis cause changes in host metabolism (Lanis et al., 2017). In this study, the faecal metabolite profiles were significantly differed between SEB and WRS groups. 26 differential metabolic pathways were detected through KEGG data analysis. Significantly different metabolites were enriched mainly in tryptophan metabolism. The microbiota composition determines the levels and nature of tryptophan catabolites, which profoundly affect aryl hydrocarbon receptors, thereby influencing epithelial barrier immunity (Morris et al., 2017). Studies have shown that there are many enrichment pathways during amino acid metabolism, such as acetate generation from acetyl-CoA I, succinic acid and propylene glycol, are related to SCFAs (mainly acetic acid and propionic acid) (Li et al., 2016; Louis and Flint, 2017; Ma et al., 2022; Zhao et al., 2022). Our results demonstrated that WRS treatment increased the acetic acid and propionic acid content in ARDS mice. Besides, WRS also increased the content of other SCFAs, such as butyrate and valeric acid, in the SEB group. SCFAs are mainly produced by gut microbiota metabolism and play an important role in regulating human health and disease (Vinolo et al., 2011; Rios-Covian et al., 2016). In particular, SCFAs could reduce the level of pro-inflammatory cytokines and inhibit immune cells’ activation, migration and proliferation. These effects of SCFAs may be attributed to the activation of FFAR2 and FFAR3, resulting in suppressing histone deacetylases, affecting energy metabolism and thus regulating inflammatory responses (Koh et al., 2016). In addition, three bioactive markers (baicalin, oroxylin A-7-O-glucuronide and skullcapflavon II) were screened out through integrated analysis of metabolite profiles and network pharmacology. These results demonstrated that WRS could ameliorate SEB-induced ARDS by altering the gut microbiota, increasing the production of microbiota-derived SCFAs and modulating the faecal metabolite profiles through the lung-gut axis.

## Conclusion

In conclusion, this study is the first to investigate the efficacy of WRS in SEB-induced ARDS mice. WRS treatment improved inflammation as well as lung tissue structure by altering the gut microbiota during SEB-induced ARDS. The anti-inflammatory activity of WRS, mainly related with the modulation of tryptophan metabolism through baicalin, oroxylin A-7-O-glucuronide and skullcapflavon II, and thus exerted a protective effect on ARDS mice. Overall, our findings demonstrate the potential of WRS as a promising candidate for the treatment of ARDS and provide novel insights into the potential mechanism of WRS in the SEB-induced ARDS mice.

## Data Availability

The datasets presented in this study can be found in online repositories. The names of the repository/repositories and accession number(s) can be found in the article/Supplementary Material.
