# Supplementary material for: Wine-processed radix scutellariae alleviates ARDS by regulating tryptophan metabolism through gut microbiota
Source: Front Pharmacol. 2023 Jan 5;13:1104280. doi: 10.3389/fphar.2022.1104280 (PMC9849372; doi:10.3389/fphar.2022.1104280)
Supplement: Supplementary file 1 [file DataSheet1.docx]

**Wine-processed Radix scutellariae alleviates ARDS by regulating tryptophan metabolism through gut microbiota**

Tingting Hu^a^*^#^* , Ying Zhu ^b^*^#^* , Jing Zhu^a^, Ming Yang ^a^, Yaqi Wang ^a*^, Qin Zheng ^a^

*^a^ Jiangxi University of Chinese Medicine, Nanchang 330004, China*

*^b^ Blood Transfusion Department, First Affiliated Hospital of Gannan Medical University, Ganzhou, 341001, China*

*^#^ These authors contributed equally to this work.*

**Supplementary Information**

**Table S1** The α-diversity values among groups (n=4-6)

| Group | Feature | ACE | Chao1 | Shannon | Coverage(%) |
| --- | --- | --- | --- | --- | --- |
| NC | 553.89±28.31 | 563.44±32.72 | 584.10±10.11 | 6.73±0.31 | 99.94±0.01 |
| SEB | 503.17±18.54** | 487.83±32.57** | 525.42±17.54**** | 6.05±0.24**** | 99.91±0.02** |
| WRS | 549.9±33.17^#^ | 552.49±38.70^##^ | 570.96±24.44^###^ | 6.56±0.20^###^ | 99.94±0.01^##^ |

SEB group compared with NC group, ***P* < 0.01, *****P* < 0.0001; WRS group compared with SEB group, ^#^*P* < 0.05, ^##^*P* < 0.01, ^###^*P* < 0.001.

**Table S2** System stability verification of QC samples (n=8)

| M/Z | RT | Intensity | Intensity(RSD%) |
| --- | --- | --- | --- |
| 688.8421 | 1.2600 | 11969.4509±15.0937 | 15.0937 |
| 589.1755 | 1.3300 | 11342.4767±12.2004 | 12.2004 |
| 603.1601 | 1.3800 | 11300.5947±9.2256 | 9.2256 |
| 603.1763 | 1.3800 | 73177.7973±7.0090 | 7.0090 |
| 675.0592 | 3.5800 | 6069.9402±11.8911 | 11.8911 |
| 636.2727 | 6.5500 | 31154.2100**±**12.2122 | 12.2122 |
| 649.2840 | 7.3000 | 22134.1323±12.0290 | 12.0290 |
| 607.3244 | 9.8600 | 16365.3334±12.4807 | 12.4807 |
| 877.3610 | 10.7200 | 987.4659±12.1903 | 12.1903 |
| 877.3831 | 10.7200 | 987.5156±12.1969 | 12.1969 |
| 605.2722 | 13.1000 | 47216.3241±8.2746 | 8.2746 |
| 580.3290 | 14.4600 | 197077.9634±23842.0757 | 12.0978 |
| 586.5176 | 24.1400 | 3428.0240±12.0287 | 12.0287 |
| 586.5046 | 24.1500 | 3428.0240±412.3481 | 12.0287 |

**Table S3** 10 chemical markers and their structures

| Peak no. | Component | Structure |
| --- | --- | --- |
| 1 | Hispidulin-7-O-glucuronide | 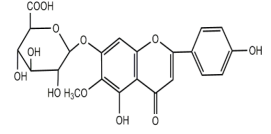 |
| 2 | Baicalin | 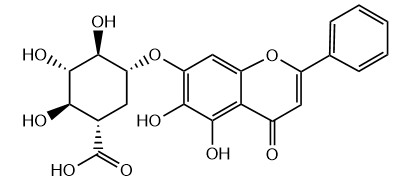 |
| 3 | Oroxylin A-7-O-glucuronide | 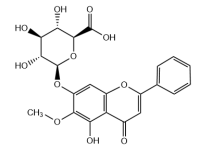 |
| 4 | Chrysin-7-O-glucuronide | 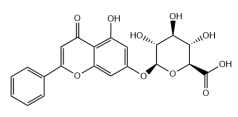 |
| 5 | Wogonoside | 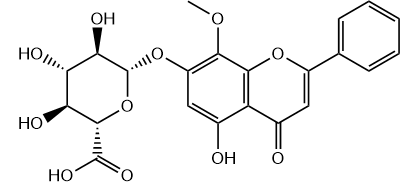 |
| 6 | Hispidulin | 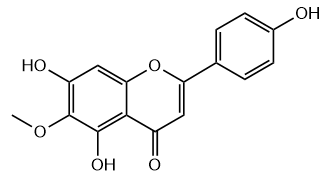 |
| 7 | Baicalein | 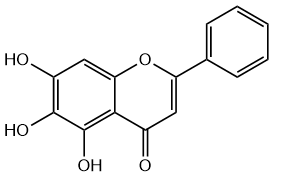 |
| 8 | Skullcapflavon II | 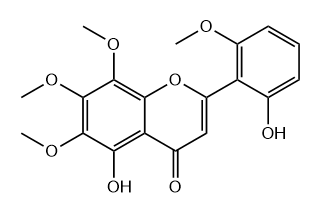 |
| 9 | Scutevulin-7-O-glucuronide | 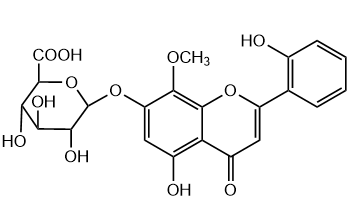 |
| 10 | Skullcapflavon I | 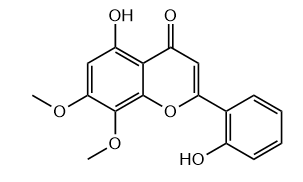 |


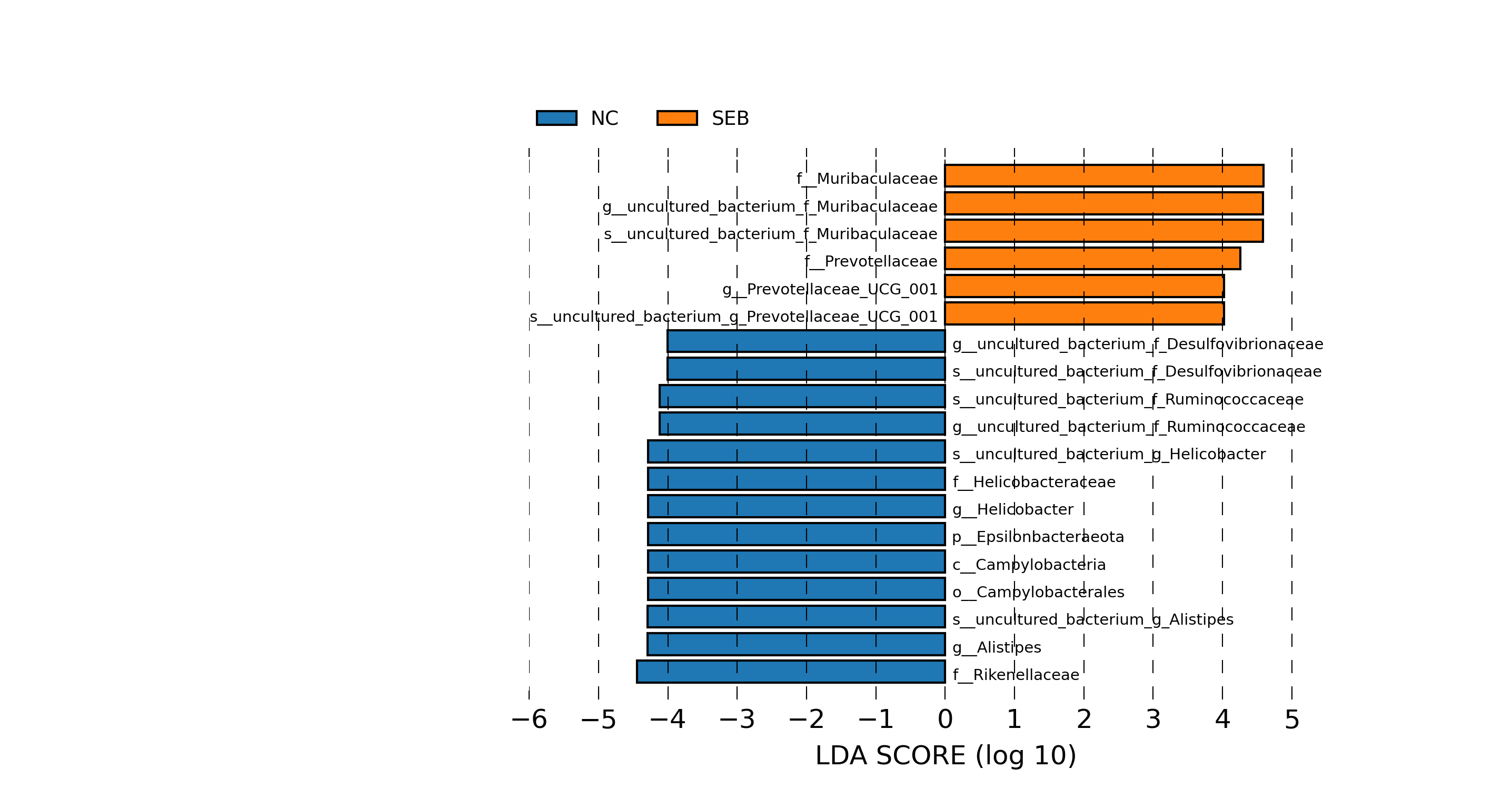


**FIGURE S1 |** LDA Score analysis between NC and SEB group.


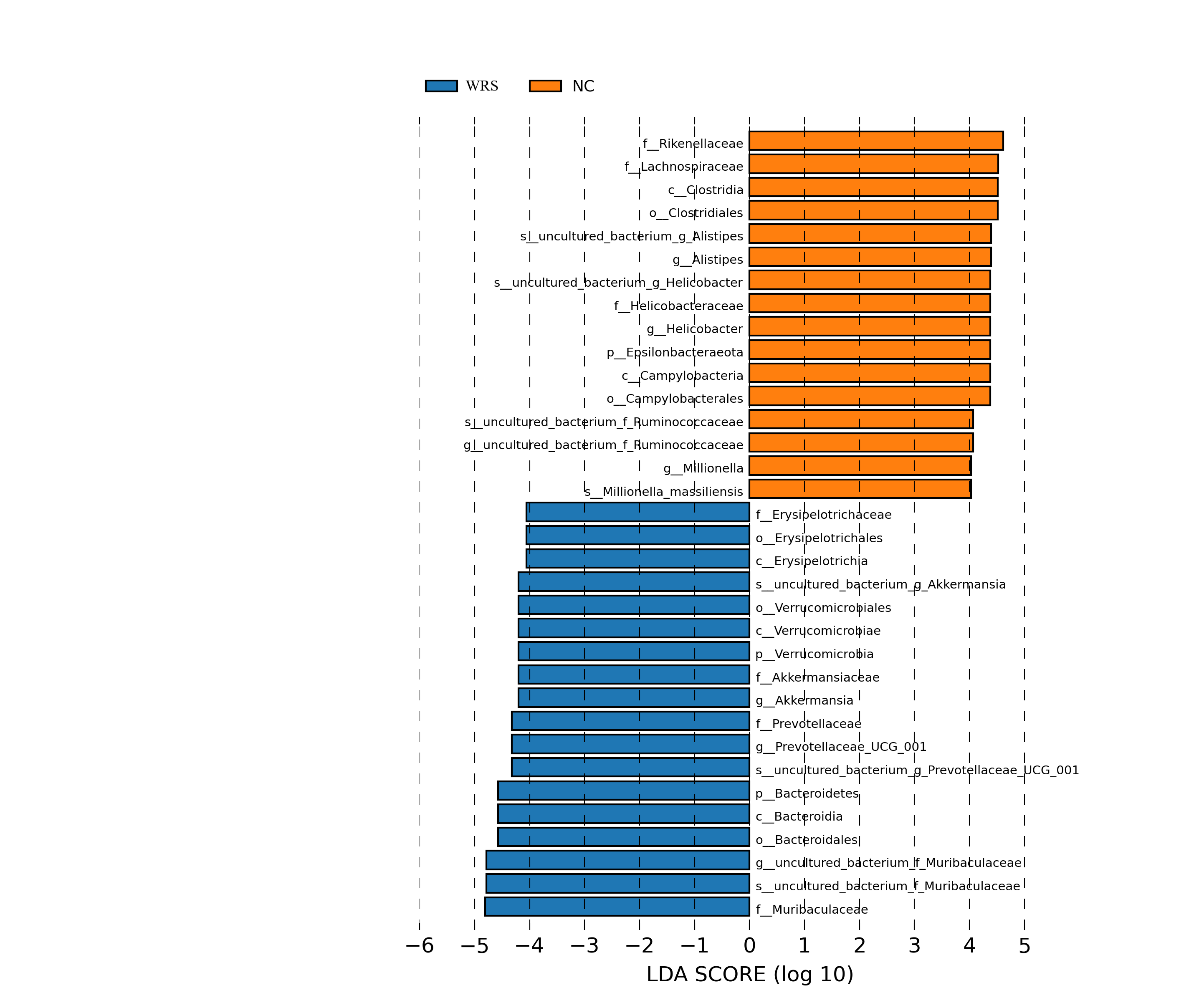


**FIGURE S2 |** LDA Score analysis between NC and WRS group.


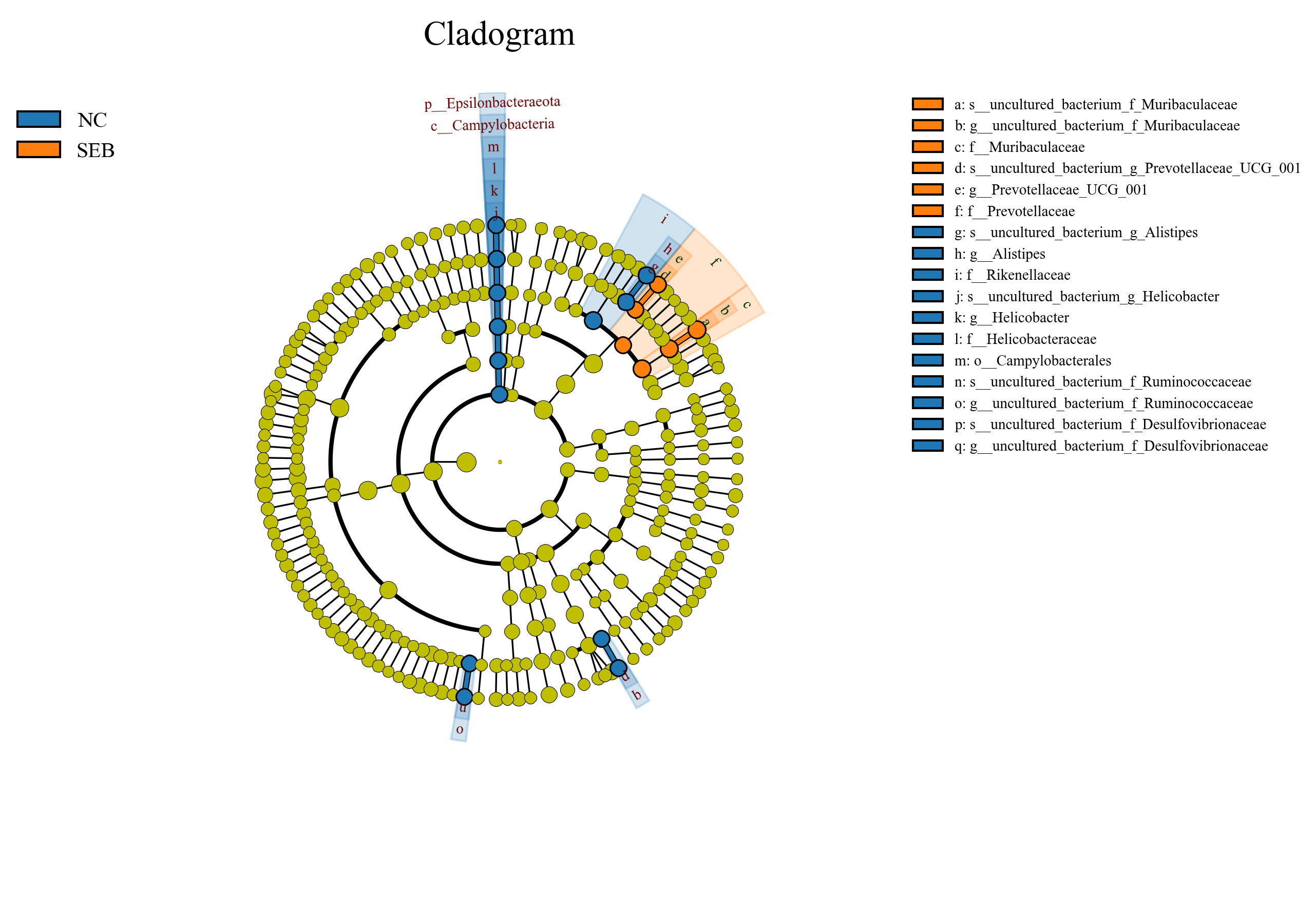


**FIGURE S3 |** Cladogram analysis between NC and SEB group.


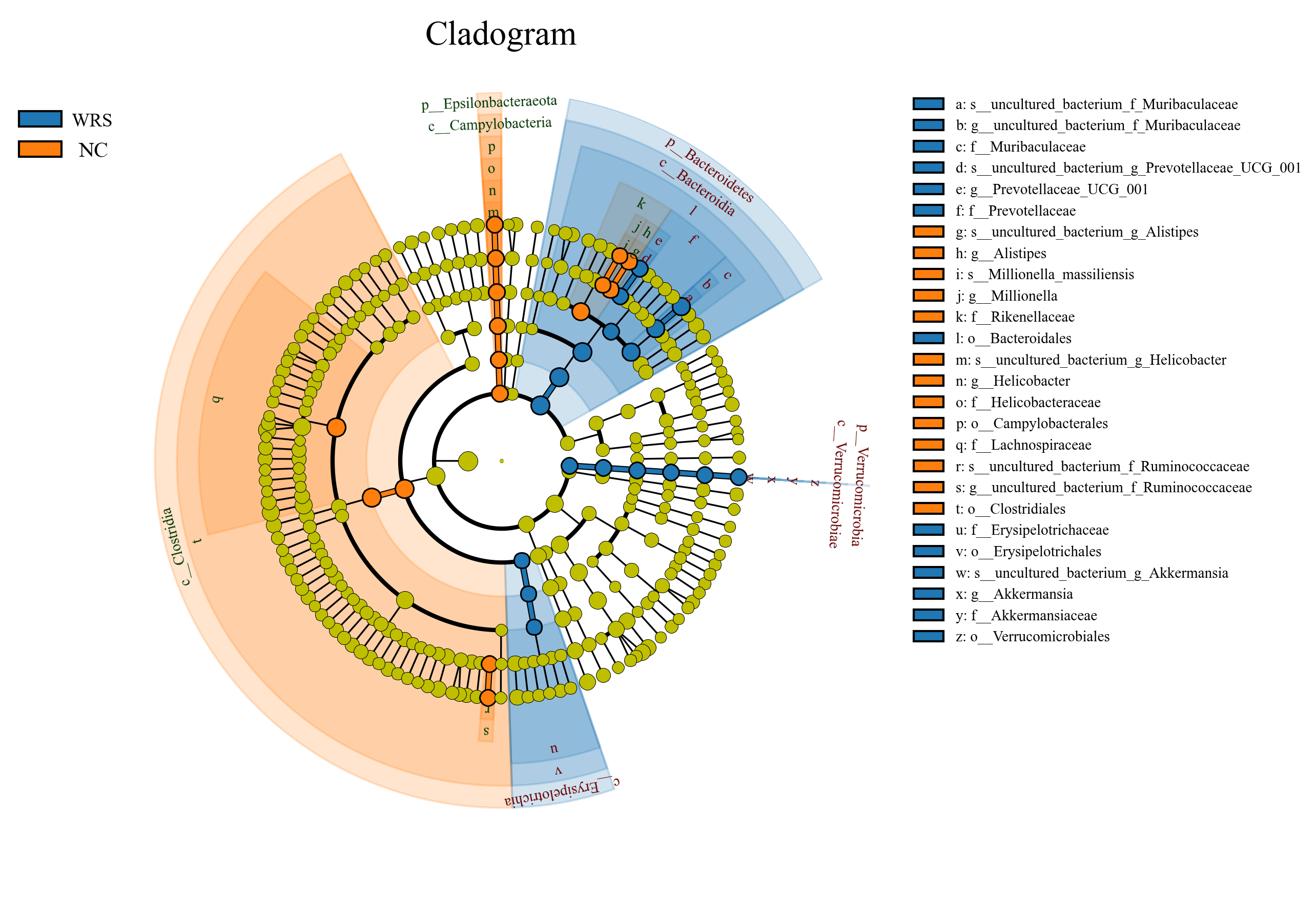


**FIGURE S4 |** Cladogram analysis between NC and WRS group.


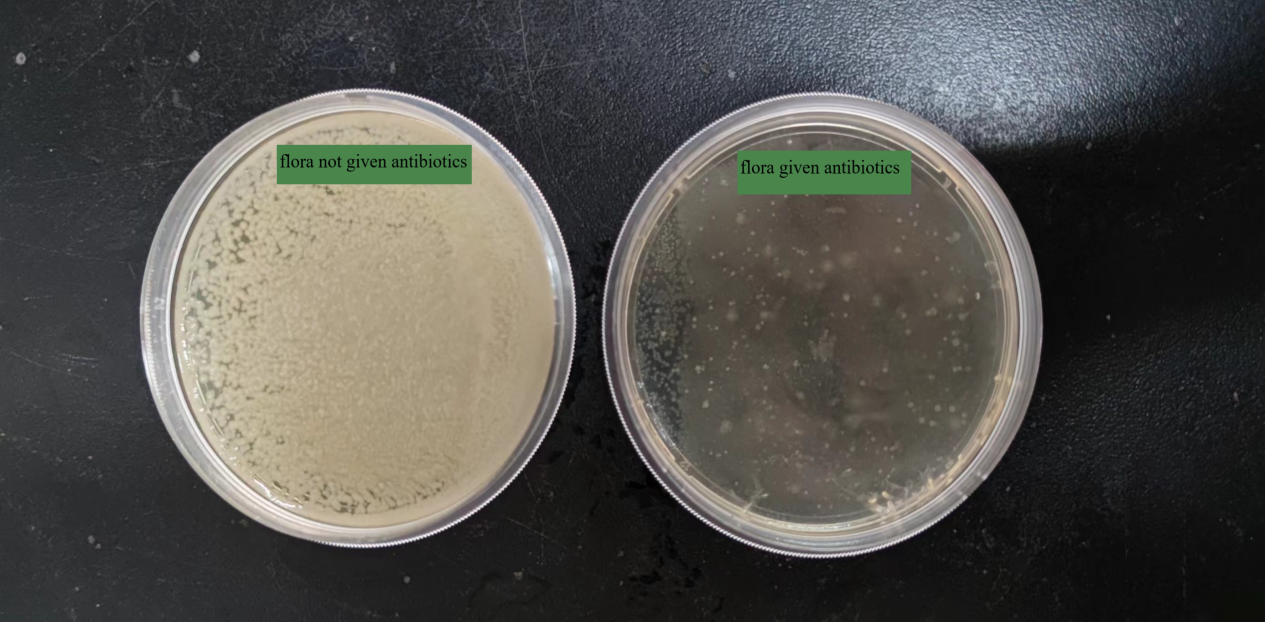


**FIGURE S5 |** The condition of gut microbes treated with or without a cocktail of antibiotics (aerobic environment).


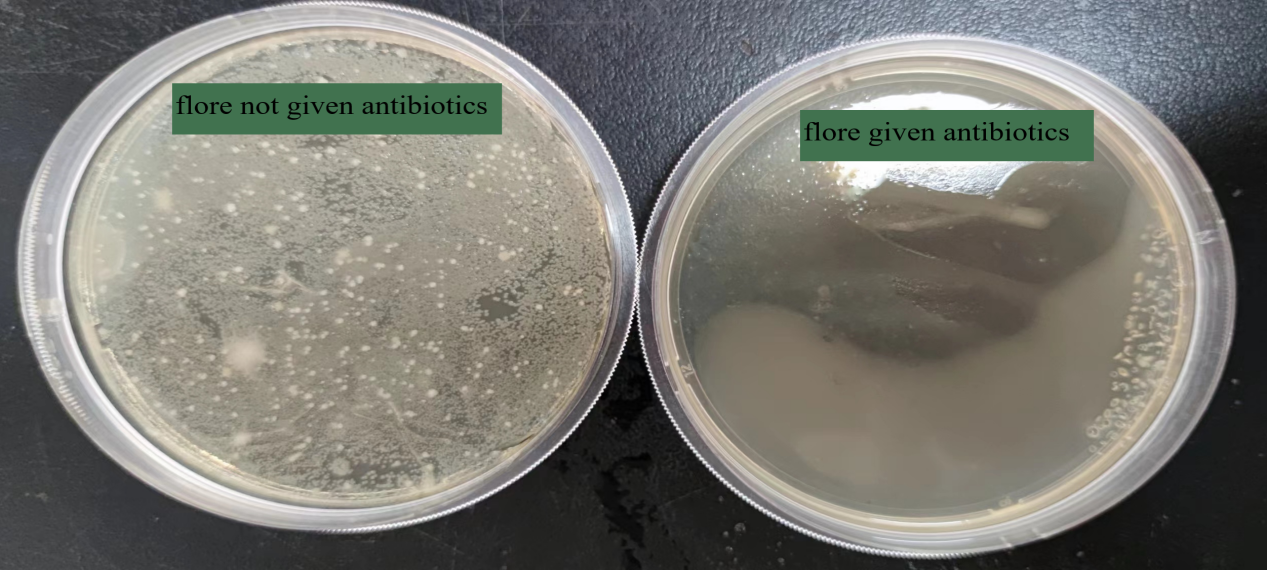


**FIGURE S6 |** The condition of gut microbes treated with or without a cocktail of antibiotics (anaerobic environment).
